# Supplementary material for: Gonadal Development and Differentiation of Hybrid F1 Line of Ctenopharyngodon idella (♀) × Squaliobarbus curriculus (♂)
Source: Int J Mol Sci. 2024 Sep 30;25(19):10566. doi: 10.3390/ijms251910566 (PMC11477168; doi:10.3390/ijms251910566)

Supplementary Materials

Table S1. Sample information.

Sample ID	Fish species	Tissue
Gc_xqn_rep1	grass carp (*Ctenopharyngodon idella*)	hypothalamus
Gc_xqn_rep2	grass carp (*Ctenopharyngodon idella*)	hypothalamus
Gc_xqn_rep3	grass carp (*Ctenopharyngodon idella*)	hypothalamus
Gc_ct_rep1	grass carp (*Ctenopharyngodon idella*)	pituitary gland
Gc_ct_rep2	grass carp (*Ctenopharyngodon idella*)	pituitary gland
Gc_ct_rep3	grass carp (*Ctenopharyngodon idella*)	pituitary gland
Gc_xx_rep1	grass carp (*Ctenopharyngodon idella*)	gonads
Gc_xx_rep2	grass carp (*Ctenopharyngodon idella*)	gonads
Gc_xx_rep3	grass carp (*Ctenopharyngodon idella*)	gonads
Sc_xqn_rep1	barbel chub (*Squaliobarbus curriculus*)	hypothalamus
Sc_xqn_rep2	barbel chub (*Squaliobarbus curriculus*)	hypothalamus
Sc_xqn_rep3	barbel chub (*Squaliobarbus curriculus*)	hypothalamus
Sc_ct_rep1	barbel chub (*Squaliobarbus curriculus*)	pituitary gland
Sc_ct_rep2	barbel chub (*Squaliobarbus curriculus*)	pituitary gland
Sc_ct_rep3	barbel chub (*Squaliobarbus curriculus*)	pituitary gland
Sc_xx_rep1	barbel chub (*Squaliobarbus curriculus*)	gonads
Sc_xx_rep2	barbel chub (*Squaliobarbus curriculus*)	gonads
Sc_xx_rep3	barbel chub (*Squaliobarbus curriculus*)	gonads
Zj_xqn_rep1	hybrid F1	hypothalamus
Zj_xqn_rep2	hybrid F1	hypothalamus
Zj_xqn_rep3	hybrid F1	hypothalamus
Zj_ct_rep1	hybrid F1	pituitary gland
Zj_ct_rep2	hybrid F1	pituitary gland
Zj_ct_rep3	hybrid F1	pituitary gland
Zj_xx_rep1	hybrid F1	gonads
Zj_xx_rep2	hybrid F1	gonads
Zj_xx_rep3	hybrid F1	gonads

Table S2. A statistical table of the number of transcripts annotated.

Annotated databases	Isoform Number
COG	43,776
GO	123,331
KEGG	93,639
KOG	110,049
Pfam	126,253
Swiss-Prot	153,110
eggNOG	148,219
nr	153,468
All	154,293

Figure S1. Differences of gonadal development-related pathways in hypothalamus (A), pituitary (B), and gonadal (C) tissues between *C. idella* and *S. curriculus*. Green indicates pathways that contain significantly down-regulated genes in *S. curriculus* compared with *C. idella*, and blue indicates pathways that contain significantly down- and up-regulated genes in *S. curriculus* compared with *C. idella*.

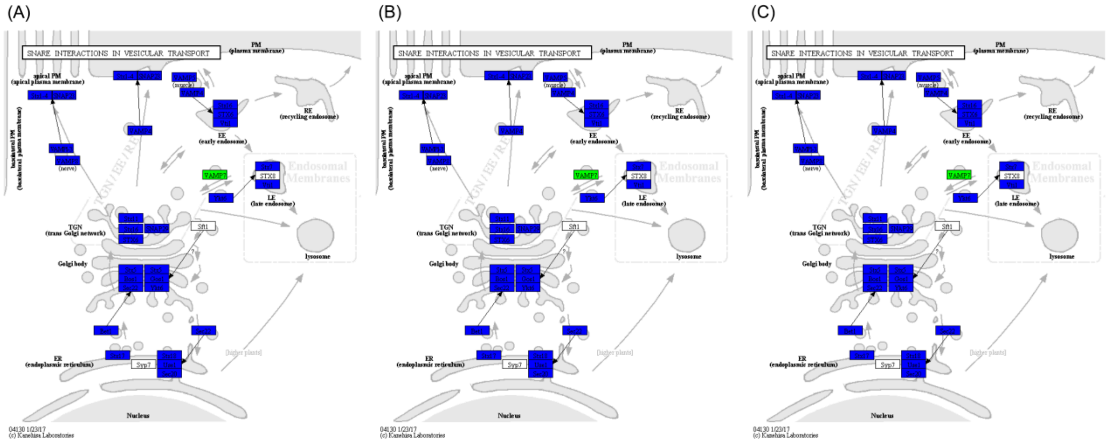

Figure S2. Differences of oxytocin signaling pathway in hypothalamus (A), pituitary (B), and gonadal (C) tissues between *C. idella* and *S. curriculus*. Red indicate pathways that contain significantly up-regulated genes in *S. curriculus* compared with *C. idella*, green indicates pathways that contain significantly down-regulated genes in *S. curriculus* compared with *C. idella*, and blue indicates pathways that contain significantly down- and up-regulated genes in *S. curriculus* compared with *C. idella*.

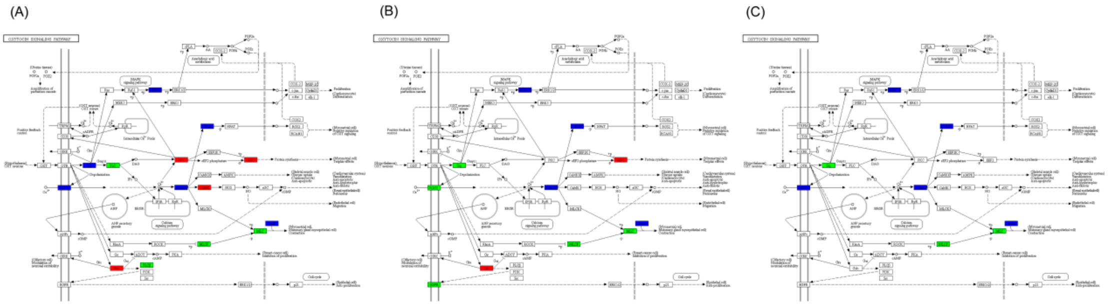

Figure S3. Differences of renin secretion pathway in hypothalamus (A), pituitary (B), and gonadal (C) tissues between *C. idella* and *S. curriculus*. Red indicate pathways that contain significantly up-regulated genes in *S. curriculus* compared with *C. idella*, green indicates pathways that contain significantly down-regulated genes in *S. curriculus* compared with *C. idella*, and blue indicates pathways that contain significantly down- and up-regulated genes in *S. curriculus* compared with *C. idella*.

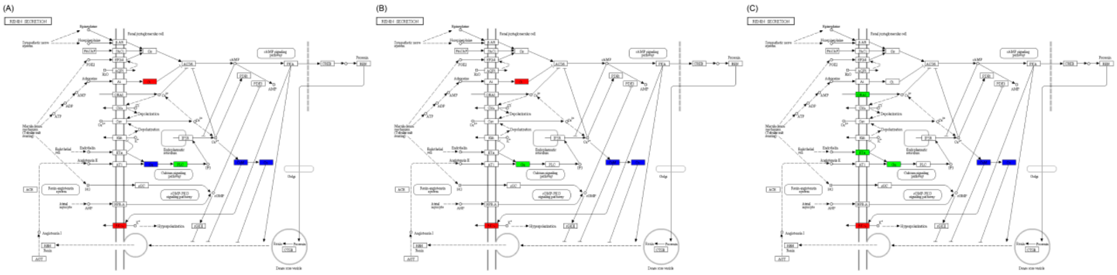

Figure S4. Differences of oocyte meiosis pathway in hypothalamus (A), pituitary (B), and gonadal (C) tissues between *C. idella* and *S. curriculus*. Red indicate pathways that contain significantly up-regulated genes in *S. curriculus* compared with *C. idella*, green indicates pathways that contain significantly down-regulated genes in *S. curriculus* compared with *C. idella*, and blue indicates pathways that contain significantly down- and up-regulated genes in *S. curriculus* compared with *C. idella*.

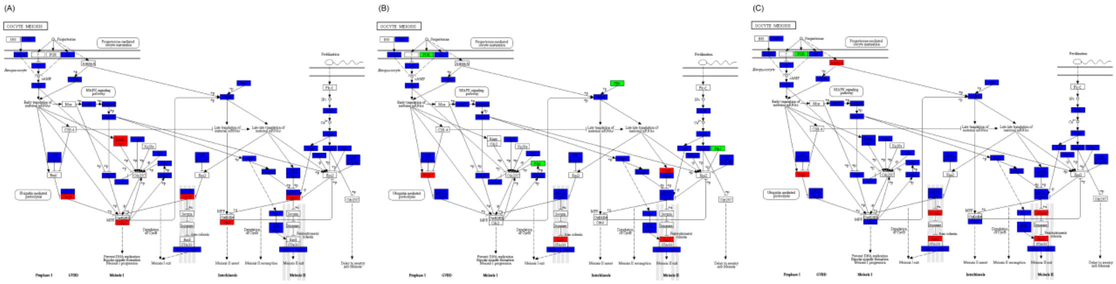

Figure S5. Differences of GnRH signaling pathway in hypothalamus (A), pituitary (B), and gonadal (C) tissues between *C. idella* and *S. curriculus*. Red indicate pathways that contain significantly up-regulated genes in *S. curriculus* compared with *C. idella*, green indicates pathways that contain significantly down-regulated genes in *S. curriculus* compared with *C. idella*, and blue indicates pathways that contain significantly down- and up-regulated genes in *S. curriculus* compared with *C. idella*.

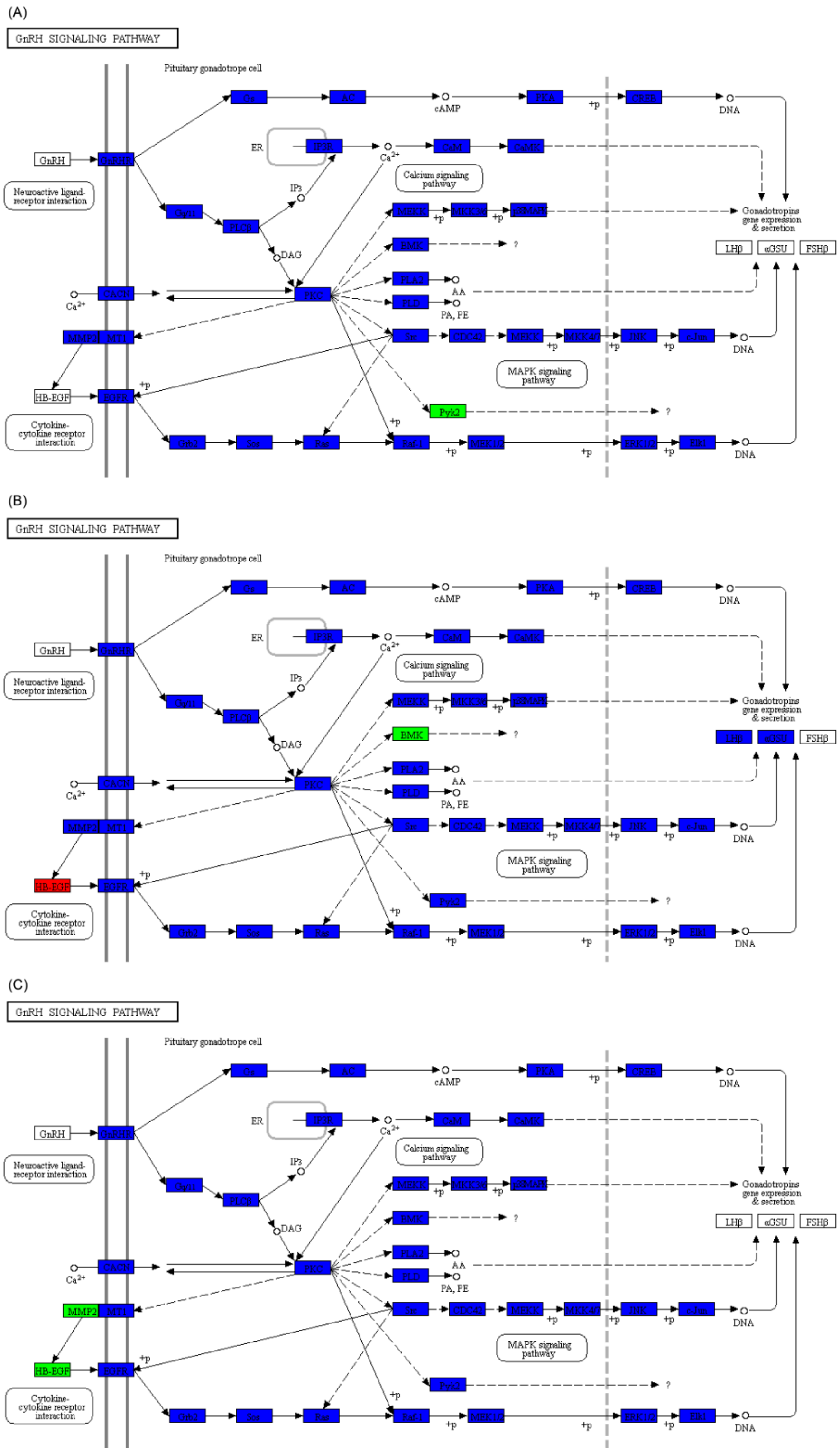

Supplement: Supplementary file 1 [file ijms-25-10566-s001.zip › ijms-3212280-supplementary.pdf]
